# Advances in Surgical Treatments of Left Ventricular Aneurysms

**DOI:** 10.31083/j.rcm2508290

**Published:** 2024-08-16

**Authors:** Jun-Hang Jia, Wei Fu, Yi-Ping Sun, Cong Chen, Kui Zhang, Ran Dong

**Affiliations:** ^1^Department of Cardiac Surgery, Beijing AnZhen Hospital, Capital Medical University, 100029 Beijing, China

**Keywords:** left ventricular aneurysm, surgical ventricular reconstruction, prognosis

## Abstract

Despite improvements in the early intervention of myocardial infarction (MI) in recent decades, left ventricular aneurysms (LVA) remain a major health concern, particularly in developing nations. The progression of MI can lead to the thinning of the myocardial wall and the formation of a ventricular wall bulge, characteristic of an LVA. Furthermore, cardiac magnetic resonance (CMR) has emerged as the gold standard for LVA diagnosis due to its superior imaging capabilities. Notably, surgical ventricular reconstruction (SVR) is an effective treatment for LVA, aiming to restore the normal volume and structure of the left ventricle, thereby improving cardiac function. However, the criteria for selecting patients for SVR treatment remains a subject of debate. This review focuses on the current understanding of surgical indications, procedures, and prognostic risk factors that influence outcomes in left ventricular reconstruction, highlighting the need for precise patient selection to optimize surgical benefits.

## 1. Introduction

Despite advancements in cardiovascular interventional therapies, left ventricular aneurysms (LVA) remains a prevalent mechanical complication following transmural myocardial infarction. Consequently, this condition can not only impair both left ventricular systolic and diastolic dysfunction, but also damage its normal structure [1], potentially leading to complications such as ventricular thrombosis, arrhythmias, and heart failure. Our understanding of LVA pathology and diagnostic approaches has deepened, with the Surgical Treatment of Ischemic Heart Failure (STICH) [2] indicating no significant benefit from adding surgical ventricular reconstruction (SVR) to coronary-artery bypass grafting (CABG). This raises important questions about the necessity and approach to LVA treatment and the identification of patients likely to benefit from specific interventions. Currently, surgery is the primary treatment for LVA. However ongoing improvements in surgical techniques, exploration of interventional strategies, and a better understanding of prognostic factors are leading to improved patient outcomes.

## 2. The Classification and Pathophysiological Mechanism of LVA

A LVA is broadly defined as a segment of the ventricular wall that exhibits dyskinetic or akinetic behavior, contributing to a reduction in left ventricular ejection fraction (LVEF) [3]. Furthermore, LVAs can be classified into two main categories: true aneurysm and pseudoaneurysm (Table 1). True aneurysms are characterized by the replacement of myocardial tissue with by scar tissue following myocardial infarction (MI), leading to dilation and deformation during cardiac cycles. In contrast, pseudoaneurysm occur following a rupture in the ventricular wall, with the resultant blood being contained by the surrounding pericardium and thrombus, thereby forming a sac that remains connected to the ventricular cavity [4]. Functional ventricular aneurysms, a subset of true aneurysms, may develop if residual myocardium within the infarcted area remains viable. These aneurysms are notable for their indistinct boundary between infarcted and normal myocardium, bulging only during systole [5]. The incidence of functional aneurysms is on the rise, attributed to early initiation of thrombolytic therapy [6] and revascularization procedures [7]. Several studies have identified transmural MI without coronary collateral circulation as a primary factor in the development of ventricular aneurysms [8, 9, 10]. Furthermore, Klein *et al*. [11] found that ventricular dilatation and ventricular wall thinning in the ischemic area occur when the infarcted myocardium accounts for more than 20% of the ventricular perimeter. This condition exacerbates ventricular wall tension and increases myocardial oxygen demand, perpetuating a harmful cycle.

**Table 1.** **Differences between true aneurysm and pseudoaneurysm**.

Differences		True aneurysm	Pseudoaneurysm
Characteristics	Width of the neck	Wide	Narrow
	Myocardial rupture	No	Yes
	The composition of the ventricular wall	Necrotic myocardium, fibrotic tissue or both	Fibrous pericardial tissue and/or blood clots
Diagnose	Echocardiography	The ventricular wall in the lesion area became thin and dilated during systole (paradoxical motion)	The echo of the ventricular wall was interrupted in the lesion area, and blood flow was observed between the aneurysm and the ventricular cavity
	Cardiac magnetic resonance	The myocardium of the lesion was thin, and a lot of scar myocardium was formed; transmural enhancement	Obliteration of myocardial-pericardial interface and pericardial enhancement
Treatment principles		Restore normal structure of left ventricular	Obliterate the neck of the pseudoaneurysm
Treatment		Surgical ventricular reconstruction or percutaneous ventricular restoration (the Parachute device, the Revivent TC System)	Surgical ventricular reconstruction or septal occluders, ventricular septal defect occluders, and coils

## 3. Imaging of LVA

Echocardiography stands as the primary diagnostic tool for ventricular aneurysms, offering detailed insights into aneurysm size, location, shape, wall motion and overall cardiac function [12]. Dobutamine stress echocardiography is also effective in identifying myocardial regions that, despite being affected by ischemia or infarction, retain the potential to recover function, known as viable myocardium [13]. Despite its widespread use, the technique’s accuracy, particularly with conventional two-dimensional ultrasound, heavily relies on the practitioner’s expertise, potentially leading to overlooked small ventricular aneurysms and mural thrombus [14]. In recent years, the development of real-time three-dimensional echocardiography (RT3DE) has notably enhanced the precision in assessing cardiac structure and function [15].

Cardiac magnetic resonance (CMR) excels in measuring cardiac volume and ejection fraction, outperforming other methods [16]. Moreover, its late gadolinium enhancement (LGE) is the gold standard for evaluating viable myocardium, surpassing dobutamine stress echocardiography and positron emission tomography and also proving effective in detecting mural thrombus [17, 18]. Additional CMR advantages include its non-invasive nature, absence of radiation, high repeatability, and superior spatial resolution, making it arguably the best imaging method for evaluating ventricular aneurysms [19]. However, longer examination times and limitations for patients with claustrophobia or ferromagnetic implants present practical challenges.

## 4. Surgical Strategy for LVA

The 2009 STICH trial [2], published in the *New England Journal of Medicine*, questioned the efficacy of SVR. While SVR significantly reduces left ventricular volume, there was no notable difference between the SVR+CABG group compared to the CABG-alone group in terms of symptom improvement, all-cause mortality, and rehospitalization for cardiovascular causes. However, the trial’s conclusions have been met with skepticism due to several limitations: (1) Patient recruitment challenges led to broadened inclusion criteria, allowing the enrollment of patients with left ventricular end-systolic volume index (LVESVI) <60% [20]. (2) The assessment of cardiac structure and function at baseline and follow-up utilized three different imaging methods: CMR, echocardiography, and computed tomography, introducing variability to the results [20]. (3) The trial did not include evaluations for viable myocardium, omitting a critical factor in determining the effectiveness of SVR [20]. (4) The multicenter nature of the study complicated the standardization of surgical approaches, a significant challenge given the intricacies of ventricular aneurysm surgery [20]. (5) The reduction rate in the left ventricular end-systolic volume was deemed insufficient, at only 19% [21]. Additionally, a retrospective analysis by Ma *et al*. [22] reported that in a cohort of 121 patients with LVA undergoing CABG-alone, patients experienced a perioperative MI and mortality rate of 1.5% and an overall 2-year postoperative patient survival rate of 91.6%. These studies suggests that CABG treatment without SVR does not contribute to the sustained expansion of LVA, supporting the feasibility of CABG as a standalone treatment for LVA.

In contrast, recent retrospective studies [23, 24, 25] have highlighted the benefits of SVR in patients with LVA, noting reduced left ventricular volume, return of normal left ventricular morphology, improved cardiac function, and reduced rates of cardiac readmission. Building on this, Yang *et al*. [26] investigated whether SVR and CABG could improve long-term outcomes in patients with ventricular aneurysm and heart failure (HF). In this study 130 patients were divided into either a combined CABG+SVR group or a CABG-alone group. Preoperative assessment of scarred myocardium and cardiac function were assessed using LGE-CMR, ensuring that both groups had comparable baseline conditions. After a median follow-up of 10 years, the CABG+SVR group exhibited a significantly lower rate of HF rehospitalization compared to the CABG-alone group (3.1% vs. 20.6%), suggesting that SVR, when added to CABG, may offer substantial long-term benefits for patients with LVA and HF.

Currently there are three main guidelines for the surgical treatment of ventricular aneurysm (Table 2). The 2022 American guidelines for the management of heart failure recommend SVR in conjunction with CABG for patients with intractable HF, large thrombus, or persistent arrhythmias caused by LVA, when other treatments fail or are not viable [27]. The 2021 American Association for Thoracic Surgery (AATS) Expert Consensus proposed that concurrent SVR with CABG is indicated for patients with no viable myocardium, dyskinesis >35% of the anterior wall, LVESVI ≥60 mL/m^2^ or an anticipated reduction in LVESVI of more than 30% through SVR [28]. This consensus is the first to use myocardial viability to assess ventricular aneurysm. However, it does not specify the location and extent of the viable myocardium. Finally, the 2018 European revascularization guidelines recommend performing SVR with CABG in patients with New York Heart Association (NYHA) functional class III/IV, large LVA or thrombus formation, arrhythmias arising from the aneurysm, and HF symptoms, with a recommended grade IIA, evidence level class C [29]. In contrast to the STICH study [2], all three guidelines or expert consensus recommended SVR+CABG as a surgical intervention for LVA.

**Table 2.** **Guideline recommendations or consensus regarding surgical treatment of ventricular aneurysm**.

Consensus or guidelines	COR	LOE	Indication	Surgical treatment
2022 AHA/ACC/HFSA Guideline for the Management of Heart Failure	-	-	intractable HF, large thrombus, or persistent arrhythmias resulting from well-defined aneurysm or scar	CABG+SVR
2021: The American Association for Thoracic Surgery Expert Consensus Document	IIa	B-R	absent viability, dyskinesis ≥35% of the anterior wall, and LVESVI ≥60 mL/m^2^, an SVR achieving a >30% reduction in LVESVI	CABG+SVR
2018 ESC/EACTS Guidelines on myocardial revascularization	IIa	C	NYHA class III/IV, large LV aneurysm, large thrombus formation, or if the aneurysm is the origin of arrhythmias	CABG+LV aneurysmectomy

COR, classes of recommendations; LOE, level of evidence; IIa, weight of evidence/opinion is in favor of usefulness/efficacy. B-R, data derived from moderate-quality randomized clinical trials or meta-analyses; C, consensus of experts and/or small studies, retrospective studies, and registries; AHA, American Heart Association; ACC, American College of Cardiology; HFSA, Heart Failure Society of America; HF, heart failure; CABG, coronary artery bypass graft; SVR, surgical ventricular reconstruction; ESC, European Society of Cardiology; EACTS, European Association for Cardio-Thoracic Surgery; NYHA, New York Heart Association; LV, left ventricular; LVESVI, left ventricular end-systolic volume index.

## 5. Surgical Treatment of LVA

For the treatment of LVA, the effect of drug therapy is limited, and the efficacy of percutaneous ventricular restoration (PVR) needs further study. At present, the primary treatment of ventricular aneurysm remains surgical operation [30]. SVR can restore the original structure and function of the left ventricle by resecting scarred myocardium, reducing left ventricular volume, and reconstructing the left ventricular conical structure [31]. Notably, the surgical treatment of LVA has more than 60 years of history [32]. The guiding principle of operation has evolved from the resection of ventricular aneurysms to the isolation of the infarct area, the reduction of left ventricular volume, and the restoration of the normal conical structure of the left ventricle [33]. The surgical procedure also developed from linear suture to patch plasty to reduce the left ventricular volume, and then to patch plasty to restore the direction of the left ventricular muscle fibers. An overview of major trials in the field can be seen in Table 3 (Ref. [32, 34, 35, 36, 37, 38, 39]).

**Table 3.** **Surgical procedures and percutaneous intervention of LVA**.

Treatment methods	Time	Number of patients	Perioperative mortality	Follow-up time	Survival rates	Characteristics
Standard linear closure (Sandwich closure) [32]	1958	81	3.7%	5 y	71%	The first SVR procedure, used for small aneurysms
Endoventricular circular patch plasty (EVCPP) [36]	1989	113	4.4%	5 y	73%	The most used SVR procedure
Septal anterior ventricular exclusion (SAVE) [37]	2006	29	0	5 y	80.3%	Emphasized reconstruction of left ventricular morphology with volume reduction
The horseshoe repair [38]	2008	15	0	6.9 m	93.3%	The absence of a patch that reduces left ventricular volume while maintaining left ventricular compliance
Keep fibers orientation with Strip patch reshaping (KISS) [39]	2009	21	0	2 y	100%	Restore the anatomic fibers’ contiguity and orientation
Percutaneous left ventricular restoration (Parachute) [35]	2011	31	0	3 y	93.5%	The most promising interventional therapy
The Revivent myocardial anchoring system (REVIVENT-TC) [34]	2018	86	4.5%	1 y	90.6%	It requires the cooperation of a cardiologist and a cardiac surgeon

SVR, surgical ventricular reconstruction; LVA, left ventricular aneurysms; y, year.

**Fig. 1.**
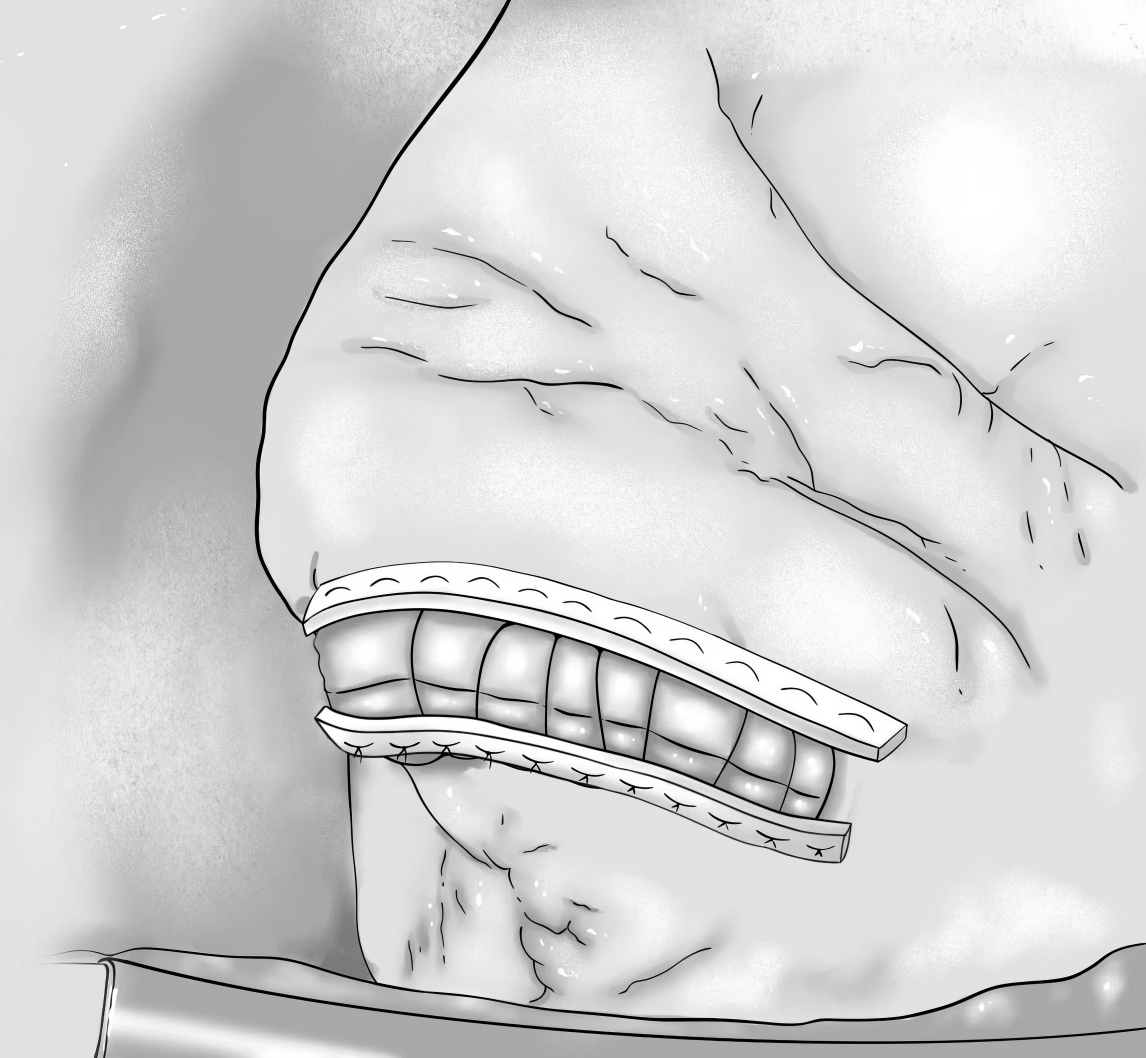
**Diagram of the Cooley technique**.

In 1958, Cooley *et al*. [32] performed the “Standard linear closure” or “Sandwich” closure of LVA under cardiopulmonary bypass (Fig. 1). The operation was conducted as follows: after cutting the aneurysm lengthwise and clearing the thrombus, the ventricular wall was removed parallel to the anterior descending artery, the scar tissue of 1–2 cm was retained and consolidated with two mats, a horizontal U-shaped suture was performed, followed by a vertical suture [32]. The technique has many advantages, including it’s simplicity and the avoidance of artificial materials in the ventricular cavity. The disadvantages include reduced functional ventricular cavity, non-geometric reconstruction, and failure to remove the infarcted portion of the ventricular septum from the cavity. The procedure is suitable for patients with a small aneurysm and a well-defined fibrous scar [40].

In 1985, Jatene proposed the concept of left ventricular geometric reconstruction, in which twisted muscle fibers are restored to their normal position and orientation [41]. The procedure consisted of a longitudinal incision of the aneurysm, folding and suturing the aneurysm of the distal ventricular septum, and a purse-string suture at the junction of normal myocardium and scar tissue. If the wound diameter is less than 3 cm, the wound is closed by linear suture. Otherwise, the wound is closed by the patch method. Jatene outlined five key considerations for ventricular aneurysm surgery. (1) The extent of the infarct size; the infarct size is decided by the blood supply area of the culprit artery, the degree of stenosis, and the degree of collateral circulation. (2) Location of the infarct area; which portion of the wall is the infarct area and whether the interventricular septal is involved make a difference in surgical strategy. (3) Expansion of the infarcted area; there is no direct relationship between the infarct size and the expansion of the infarct area. (4) The state of the non-infarct area; the non-infarct area of the myocardium will directly affect the wall stress and overall ventricular systolic function. (5) Complications of the aneurysm; these include various arrhythmias, intraventricular thrombi, and papillary muscle dysfunction. Of the 508 patients who underwent the Jatene procedure in 1977–1983, the perioperative mortality was 4.3%, and the long-term mortality was 3.5%.

**Fig. 2.**
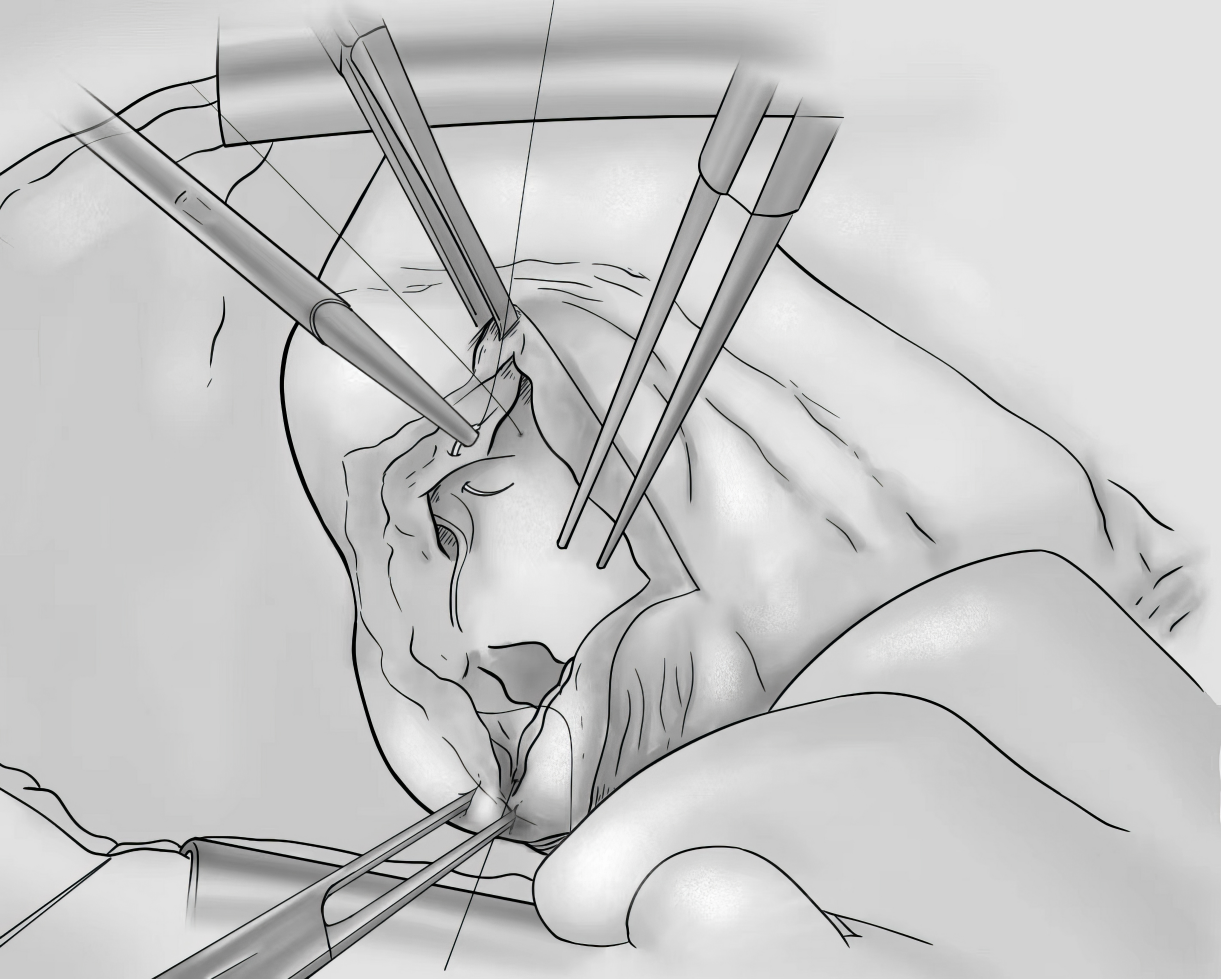
**Diagram of the Dor procedure**.

In 1989, Dor *et al*. [36] used an “Endoventricular circular patch plasty” (EVCPP) to treat ventricular aneurysms (Fig. 2). This technique involves the removal of a dyskinetic or akinetic myocardium, the fixation of a Dacron patch lined with a pericardium at the junction of viable and scar myocardial tissue, and simultaneous revascularization. This approach has several noted advantages: (1) It leaves the infarcted myocardium of the interventricular septum out to avoid its abnormal motion. (2) It reconstructs the left ventricular chamber in a normal state utilizing patch plasty, avoiding the disadvantage of a small left ventricle volume caused by linear suture technique after resectioning a giant aneurysm. (3) It preserves the anterior descending artery for bypass. (4) It does not need external prosthetic material, avoiding pericardial adhesions.

The Dor procedure is still widely used today, and the surgical approach continues to evolve and improve. Menicanti L and Di Donato [42] enhanced this technique using preshaped elliptical balloons to control the postoperative volume of the left ventricle. This modification, along with transitioning from not using an intraventricular patch to the use of a patch, has led to improved patient outcomes [43]. Research indicates that strategies focusing on restoring the left ventricle’s shape rather than merely reducing its volume yield greater benefits for patients [44, 45]. Furthermore, the surgical goal has been refined to not only reconstruct the ventricle’s conical structure but also to reduce the LVESVI to below 70 mL/m^2^ [46].

**Fig. 3.**
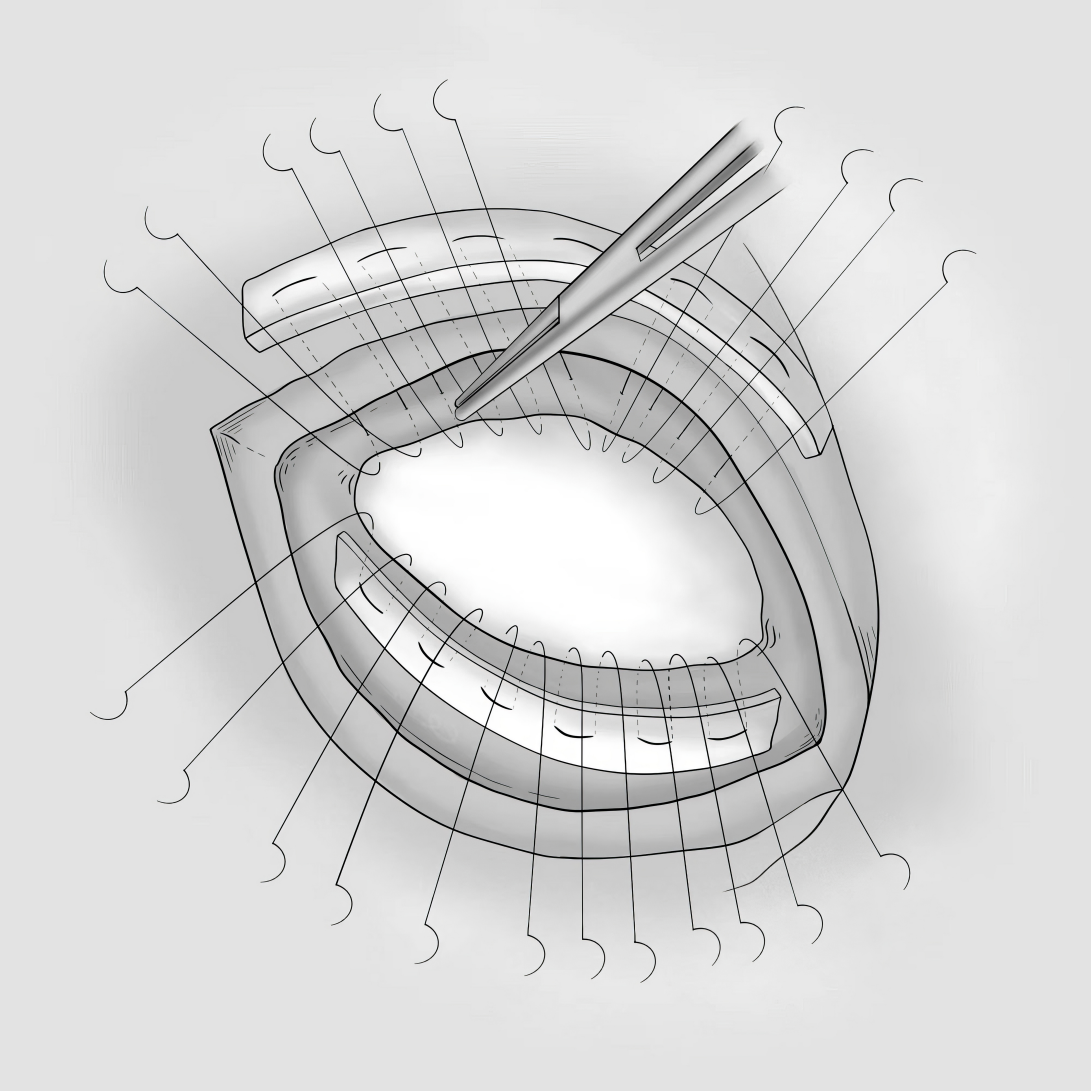
**Diagram of the “Pacopexy” procedure**.

In 2006, Isomura *et al*. [37] published the results of the septal anterior ventricular exclusion (SAVE), or “Pacopexy” procedure for ischemic cardiomyopathy (ICM) (Fig. 3). This method reconstructs the left ventricular chamber by suturing numerous mattress stitches along the exclusion line, extending from the apex to a septal site 1 cm below the aortic valve. While the Dor procedure emphasized the exclusion of the scarred myocardium and volume reduction, the SAVE procedure prioritized the reconstruction of left ventricular morphology with volume reduction. Postoperative left ventriculograms reveal that the SAVE procedure results in a more elliptically shaped left ventricle, contrasting with the more spherical shape seen after EVCPP. In particular, the study showed no difference in perioperative mortality with the SAVE procedure compared with the Dor procedure, with a 5-year survival rate of 80.3% in 29 patients after SAVE and 77.4% in 54 patients after the Dor procedure. Another recent study compared the long-term outcomes of patients after the Dor procedure and the SAVE procedure. The 10-year survival rates in the two groups were 70.4 ± 7.9% vs. 41.7 ± 7.2% (*p* < 0.05), respectively, and the rehospitalization rates for HF and cardiac death were 60.0 ± 8.6% vs. 28.8 ± 6.8% (*p* < 0.05), respectively [47]. In contrast to the original study, the updated data clearly shows the clinical efficacy of the Pacopexy treatment.

In 2008, Ferrazzi introduced “the horseshoe repair” technique for treating ventricular aneurysms, a technique characterized by the absence of a patch, which results in a decrease to the left ventricular volume while maintaining left ventricular compliance [38]. The procedure involves the placement of two horseshoe-shaped, semicircular, parallel purse strings into the scar tissue along the interventricular septum, one in the middle of the anterior papillary attachment and the other shifted 1 cm toward the apex. The first purse string is secured to achieve a conical shape, and the second is tightened to reshape the new apex. Importantly, the operation can reduce the transverse diameter of the left ventricle without shortening the longitudinal length. Furthermore, a study of 15 patients undergoing the horseshoe repair showed a survival rate of 93.3% at 6.9-months after the operation.

**Fig. 4.**
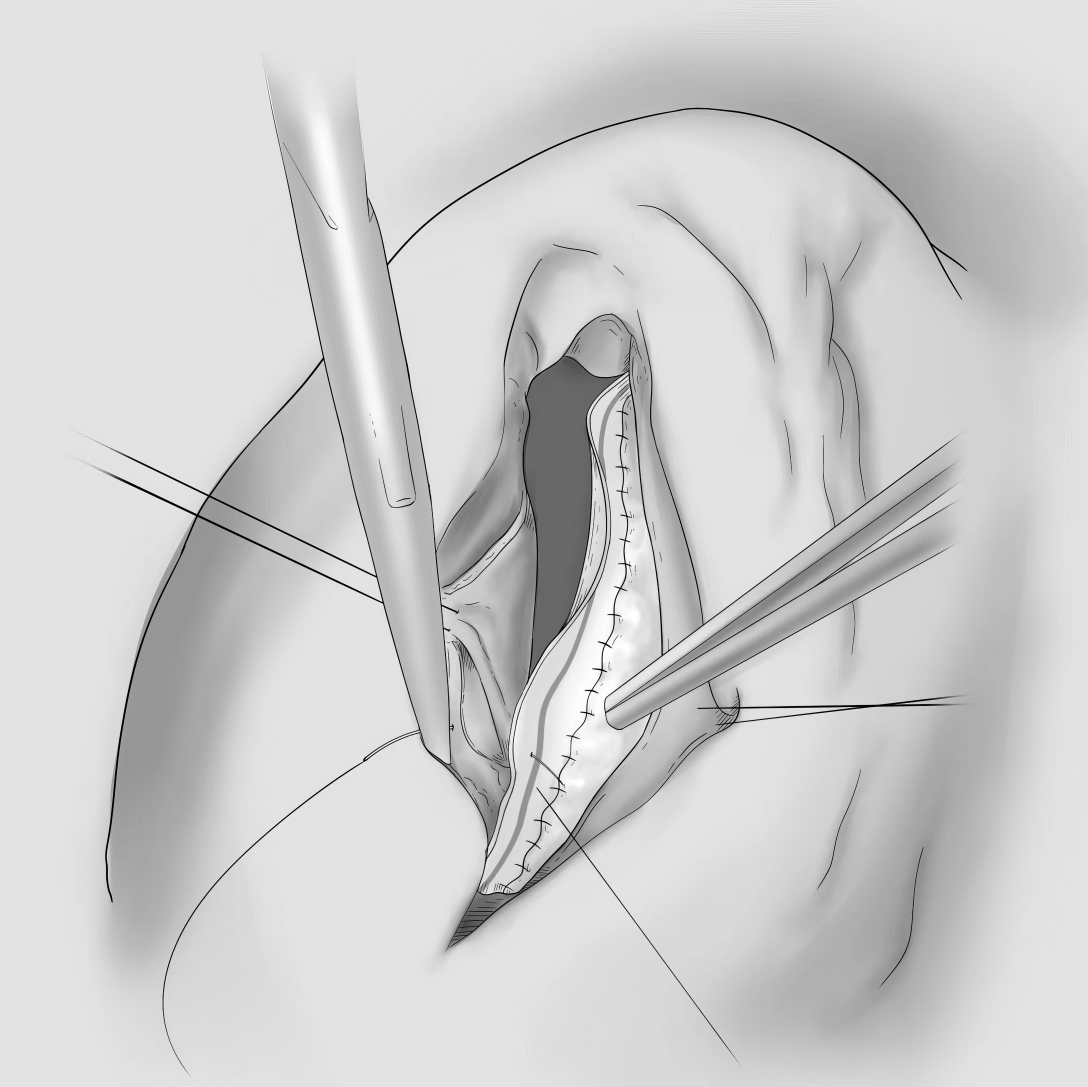
**Diagram of the KISS procedure**. KISS, Keep fibers orientation with Strip patch reshaping.

In 2009, Cirillo [39] adopted the “Keep fibers orientation with Strip patch reshaping” (KISS) procedure to restore the anatomic fibers’ contiguity and orientation, thus improving the contractile function of ischemic myocardium (Fig. 4). The key differences in the KISS procedure include: (1) utilization of a narrow and long patch with arrow-shaped ends; (2) elimination of the need for purse strings; (3) asymmetric suturing of the patch inside the ventricle. The length of the arrow-shaped patch is tailored to span from the site of the apical infarction to the aortic valve. The interventricular septum edge of the patch is sutured equidistantly along the basal border of the fibrotic septum (patch-tissue equivalence), and the lateral edge of the patch is sutured to the lateral wall at an unequal distance along the border of the normal and infarcted myocardium (patch-tissue mismatch). This procedure was performed on twenty-one patients with a follow-up period of 2 years, with all patients surviving, and a decreased LVESVI from 87.5 ± 27.9 mL/m^2^ to 35.2 ± 16.1 mL/m^2^. Left ventricular torsion, an essential parameter for assessment of left ventricular ejection function, increased from 2.5 ± 4.6 to 2.5 ± 4.6, indirectly suggesting that the myocardial bundle returned to normal physiological orientation after the KISS procedure.

In addition to surgical methods, interventional techniques have been explored to treat ventricular aneurysms. In 2007, Otasevic *et al*. [48] pioneered the first percutaneous left ventricular restoration utilizing a parachute-like device known as the left ventricular partitioning device (VPD). This device is introduced into the left ventricle was expanded with a balloon dilator, isolating the akinetic or dyskinetic myocardium and subsequently reducing the left ventricular volume. The Parachute V trial, a prospective, multi-center, post-market, non-randomized, nested-control, observational study of the parachute implant system was unfortunately terminated in June 2017 [48].

**Fig. 5.**
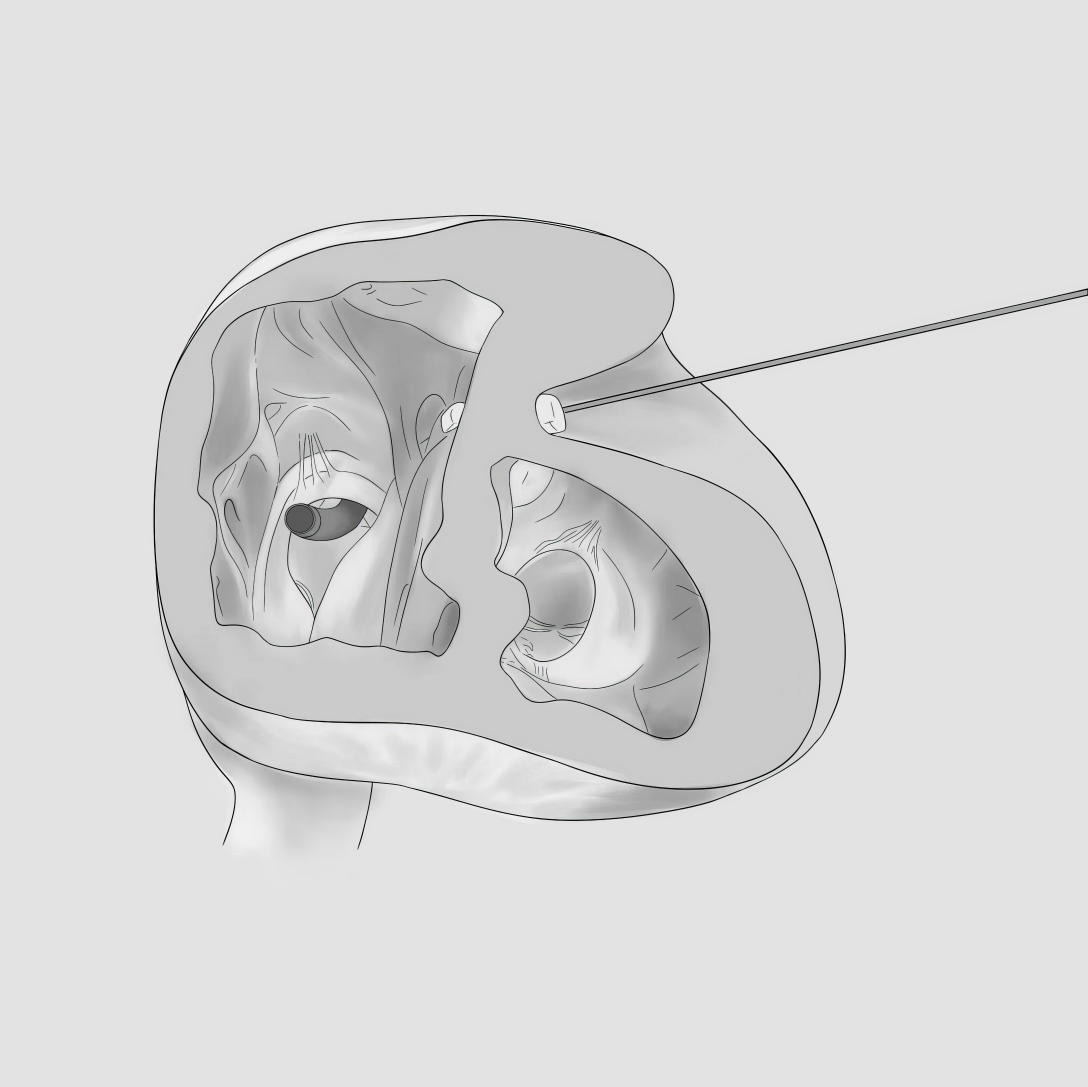
**Diagram of the Revivent TC System**.

Subsequently, the Revivent myocardial anchoring system was developed by US company BioVentrix. This consists of two anchoring devices and a connecting wire, allowing the exclusion of the aneurysm area by anchoring and drawing the anterior and septal walls together [49]. An updated version, known as the Revivent TC System, allows a less invasive implantation on the beating heart without needing a thoracotomy and cardiopulmonary bypass (Fig. 5) [34]. However, the safety and efficacy of the device still need to be confirmed by a large prospective randomized controlled trial. Another innovative approach involves combining the left ventricular assist device (LVAD) HeartMate 3 (Abbott) with a “double patch technique” in SVR. This technique can be used in patients with end-stage HF and LVA who are ineligible for heart transplantation [50].

Ventricular dysrhythmia is a major cause of reduced quality of life and elevate the risk of sudden cardiac death in individuals with LVA. However, complete resection of the aneurysm or SVR can reduce the risk of postoperative ventricular dysrhythmias in most patients [51, 52]. Methods for diagnosing ventricular dysrhythmias include signal-averaged electrocardiography (SAECG), Holter monitoring, and epicardial and endocardial mapping [53, 54]. Treatment options include implantable cardioverter-defibrillators, ventricular tachycardia (VT) ablation, and percutaneous ablation [55].

## 6. Prognostic Factors for SVR

Since the introduction of SVR, there has been a concerted effort to discern the factors influencing the prognosis of patients undergoing SVR, with the objective of pinpointing candidates most likely to benefit from the procedure. Currently, the most extensively studied factors include LVEF, LVESVI, NYHA class, mitral regurgitation (MR) grade, and restrictive filling pattern (RFP).

### 6.1 LVEF

The RESTORE (Reconstructive Endoventricular Surgery, returning Torsion Original Radius Elliptical shape to the left ventricle) group experience published in 2004 identified that an LVEF ≤30% was a risk factor for postoperative mortality in patients experiencing congestive HF following an anterior myocardial infarction [56]. Further supporting this, research by Wakasa *et al*. [57] pinpointed postoperative LVEF as a critical determinant of long-term survival, indicating that SVR+CABG improved survival benefits by increasing LVEF. Specifically, in patients with a postoperative LVESVI between 40–80 mL/m^2^, each 3.1% increase in LVEF was associated with a 21% reduction in mortality [57]. In 2023, Ma *et al*. [22] found that preoperative ventricular thrombosis and a LVEF <40% increased the incidence of major adverse cardiac and cerebrovascular events (MACCE), underscoring the significance of LVEF as a prognostic factor in the surgical treatment of heart failure.

### 6.2 LVESVI

The LVESVI is the most well-studied factor and has the closest relationship with patient prognosis following SVR [57]. The RESTORE study, not only verified the safety and efficacy of SVR surgery, but also served as a foundation for the design of the subsequent STICH trial [56], pinpointed an LVESVI ≥80 mL/m^2^ as a risk factor for postoperative mortality [56]. Furthermore, Suma* et al*. [58] also concluded that preoperative high LVESVI was a mortality risk factor, while age, inotropic drugs, and pulmonary hypertension were not statistically impactful.

A later subgroup analysis of the STICH trial published in 2009 demonstrated a distinct survival benefit from SVR+CABG over CABG alone in patients with a postoperative LVESVI ≤70 mL/m^2^ [59]. In 2010, Di Donato *et al*. [21] found increased mortality rates in patients with a postoperative LVESVI >60 mL/m^2^, consistent with the findings of Witkowski *et al*. [60]. In 2011, Skelley *et al*. [61] found that SVR successfully increased EF and relieved symptoms in patients with severe congestive HF, particularly noting that patients with a preoperative LVESVI between 80 and 120 mL/m^2^ may be ideal candidates for SVR procedures.

In the same year, a study by Isomura* et al*. [62] analyzed the relationship between the volume reduction rate after SVR and the long-term prognosis of ICM, concluding that SVR is most effective when the LV volume was decreased by more than 33% and LVESVI was brought down less than 90 mL/m^2^. Furthermore, in 2020, Cui *et al*. [47] found that a preoperative LVESVI ≥60 mL/m^2^ and an age ≥65 years were factors associated with increased risks of cardiac readmission and death. In conclusion, these findings underscore the critical role of high preoperative LVESVI or suboptimal volume reduction during SVR as risk factors marking a poor prognosis.

### 6.3 NYHA Functional Class

Several studies [23, 56, 63, 64] have identified that diagnosis with NYHA functional class III or IV is a risk factor for patients undergoing surgical interventions. Further work by Witkowski *et al*. [60] determined that advanced HF status was associated with increases in mortality and HF hospitalization rates during a 2-years of follow-up period following SVR.

### 6.4 Mitral Regurgitation Grade

Several studies have highlighted an MR of grade 2 + as a significant postoperative risk factor [22, 23, 65], with evidence suggesting that an MR ≥3 + is a risk factor for postoperative mortality [58]. Moreover, Sartipy *et al*. [66] extended this by demonstrating a direct correlation between the severity of MR and increased risk of long-term mortality and hospital readmission due to heart failure. Currently, most doctors use mitral valvuloplasty plus SVR to treat moderate to severe MR with LVA [67]. However, a study by Yin [68] in 2016 showed that patients with LVA and MR ≥3 + undergoing concurrent mitral valve repair did not experience a reduction in long-term mortality or the incidence of MACCE.

### 6.5 Diastolic Dysfunction 

Diastolic dysfunction usually manifests as an increase in early to late diastolic filling pressure >2 or restrictive filling pattern (RFP) [69]. In 2007, the Italian scholar Menicanti *et al*. [23] first proposed RFP as a significant predictor of mortality following SVR. Subsequently in 2017, Furukawa *et al*. [70] supported these findings with results indicating that RFP was the only meaningful predictor of MACCE in patients with ICM undergoing SVR. Similarly, Marui *et al*. [71] found that preoperative RFP was a major predictor of cardiovascular mortality. Crucially, Fantini *et al*. [72] found that RFP could be ameliorated in approximately 50% of patients following SVR, which was associated with a thicker left ventricular posterior wall and higher relative wall thickness (RWT) prior to surgery. In 2022, Toso *et al*. [73] stratified the prognosis of patients with a biological marker’s level and RFP. From this study, he found RFP is a risk factor for poor prognosis in patients with ICM.

Beyond the previously discussed factors, several other indicators are associated with the prognosis of patients undergoing SVR. In 2017, Couperus* et al*. [74] identified preoperative right ventricular systolic dysfunction as an independent predictor of increased mortality in patients with ICM who underwent SVR. That same year, Yang *et al*. [75] found that patients with 6 or more scarred myocardial segments had a higher risk of all types of cardiovascular events. Additionally, Adhyapak *et al*. [76] suggested that a smaller RWT often indicates dilated left ventricular (LV) remodeling, which correlates with increased mortality and a higher risk of heart failure readmission in patients with ICM. Furthermore, Choi *et al*. [77] found that a lower preoperative sphericity index (SI) was associated with better survival following SVR. Moreover, SI continued to increase despite improved LVEF and LV volume reduction suggesting a complex relationships between LV morphology, function, and post-SVR patient prognosis.

## 7. Conclusions

With advancements in early percutaneous intervention, the incidence of LVA is expected to decline, yet it remains a significant health concern. Echocardiography stands out as the most accessible diagnostic tool for LVA, while CMR provides the most accurate diagnosis. The decision to combine SVR with CABG in patients with coronary artery disease and LVA is still a subject of debate. Nonetheless, accumulating evidence suggests that surgery is beneficial for a specific subset of patients with LVA. It is essential to identify patients who can benefit from SVR, as it will inform new surgical guidelines based on the prognostic factors influencing patient outcomes.

The evolution of our understanding and treatment strategies for ventricular aneurysms continue to grow. From the initial resection of the aneurysm to the implementation of SVR, which aims not just to reduce the left ventricular volume but also to restore the normal ventricle and orientation of the muscle fibers. Additionally, the exploration of minimally invasive interventional devices through clinical trials represents the forefront of innovation in this field.

Much of the recent research consists of retrospective studies, and the few randomized controlled trials that exist face challenges due to variability in SVR procedures and small patient populations. This highlights the ongoing need for well-designed, large-scale, multicenter, randomized controlled trials employing standardized surgical approaches to further refine and validate the best treatment protocols for patients with LVA.
